# The Mysterious Mitral Mass: A Case of Valvular Myxoma

**DOI:** 10.1155/2018/3927948

**Published:** 2018-07-19

**Authors:** Ramy Mando, Julian J. Barbat, Alessandro Vivacqua

**Affiliations:** ^1^Department of Internal Medicine, Beaumont Health System, Royal Oak, MI, USA; ^2^Department of Cardiovascular Medicine, Beaumont Health System, Royal Oak, MI, USA; ^3^Department of Cardiothoracic Surgery, Beaumont Health System, Royal Oak, MI, USA

## Abstract

Myxomas are the most common benign cardiac neoplasms in adults. The vast majority of cardiac myxomas arise from the left atrium near the fossa ovalis of the intra-atrial septum. There have been reports of myxomas arising from the ventricles accounting for about 5% of cases. In our literature review, we have found 55 reported cases of myxomas originating from the mitral valve reported in the adult population dating back to 1871. The majority of these cases presented with embolic complications or syncope. We present an incidental mitral valve myxoma which we excised in efforts to prevent debilitating complications.

## 1. Introduction

Myxomas are the most common primary heart tumors accounting for 40–50% of primary cardiac tumors. These neoplasms are typically located in the left atrium; however, there have been cases of ventricular myxomas and, very rarely, valvular myxomas [1–3]. These lesions are usually benign and require surgical management. Complications associated with these masses may include those related to outflow obstruction and those related to pulmonary or systemic embolization [4, 5]. These tumors are typically sporadic although there does exist an autosomal dominant transmission in Carney's syndrome. Carney's syndrome is thought to account for about 7% of myxomas and is believed to be related to abnormalities in the second chromosome [6]. Other manifestations of Carney's syndrome include cutaneous myxomas, hyperpigmentation of the skin, and endocrinopathies [7]. We hope to further highlight other features of myxomas, methods of diagnosis, and management within this script.

## 2. Case Description

A 53-year-old female was admitted for evaluation of flank pain radiating to the left lower quadrant of her abdomen. Relevant past medical history includes previous left renal calculi requiring ureteral stenting and nonischemic cardiomyopathy with reduced ejection fraction. Laboratory studies were remarkable for leukocytosis and acute renal injury. Imaging studies revealed multiple adjacent obstructing calculi in the mid left ureter causing moderate left-sided hydronephrosis. Patient was boarded for emergent cystoscopy and underwent left ureteral stent placement with no intraoperative events.

**Figure 1:**
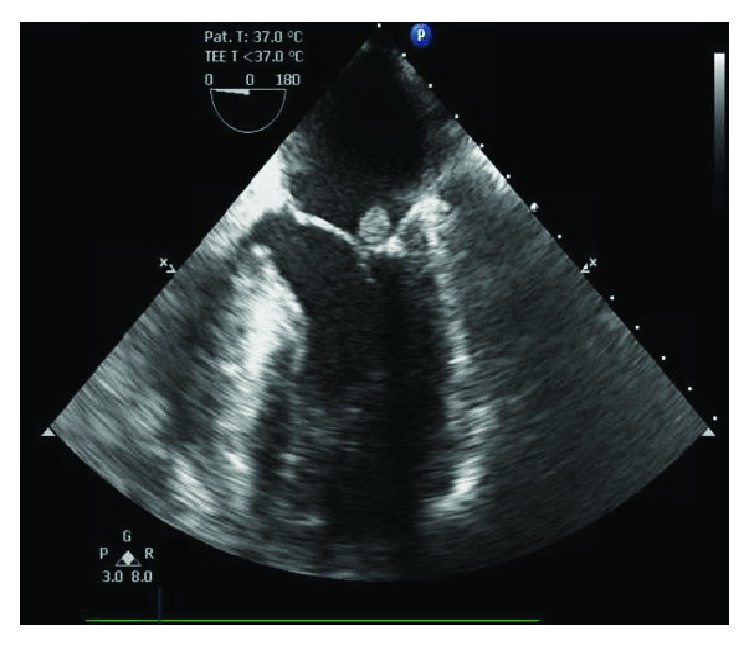
Echocardiogram imaging of a globular mass attached to the mitral valve of heterogeneous echogenicity.

**Figure 2:**
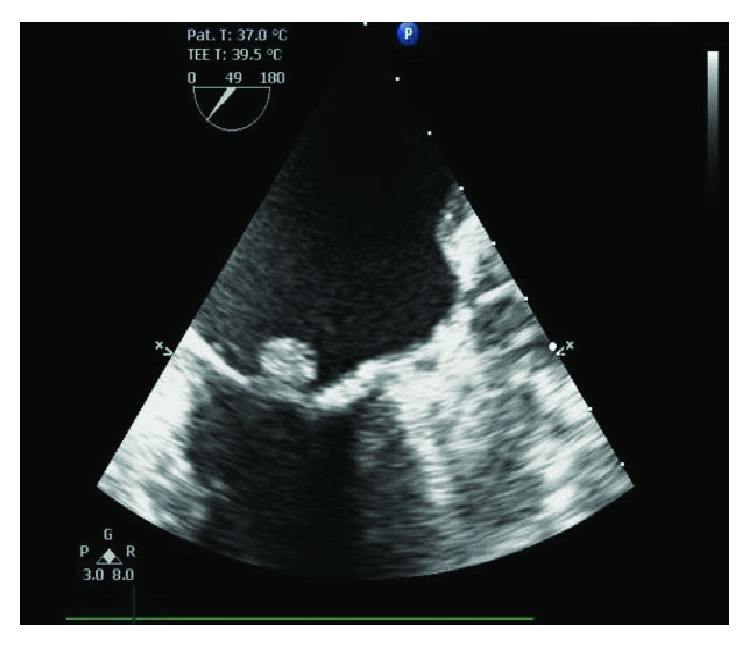
Echocardiogram imaging of a globular mass attached to the mitral valve of heterogeneous echogenicity.

Given the patient's history of cardiomyopathy, she underwent preoperative cardiac evaluation revealing a 10 × 10 mm mitral valve “vegetation” on transthoracic echocardiogram. Initial concern was for endocarditis, and the patient was started on antibiotic therapy. However, blood cultures obtained on admission remained free of microbial growth and the patient exhibited no symptoms consistent with overt endocarditis. A transesophageal echocardiogram done to better delineate the consistency of the lesion revealed a 10 × 7 mm noncalcified mass with uniform echodensity located on the atrial side of P2 (Figures 1 and 2). Differential diagnosis at this time included myxoma, papillary fibroelastoma, liposarcoma, and less likely, an infectious vegetation. Left heart catheterization revealed nonobstructive coronary artery disease and mild mitral regurgitation.

**Figure 3:**
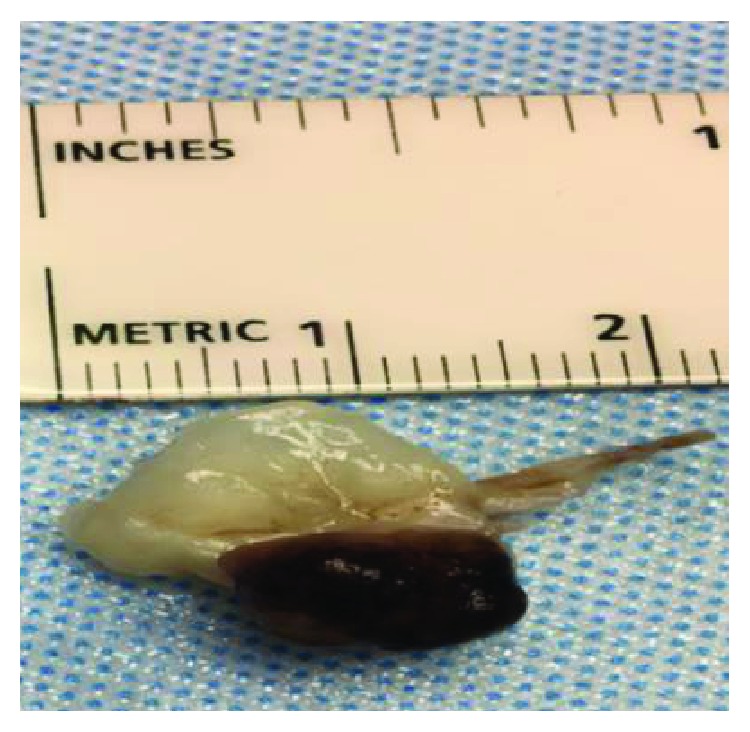
Gross pathology of the 9.0 × 8.0 × 6.0 mm myxoma.

**Figure 4:**
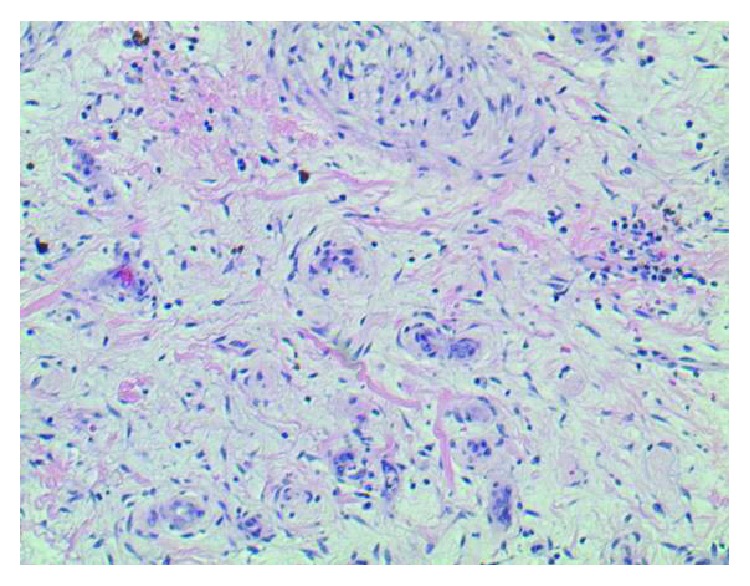
Presence of stellate cells in the background of a myxoid substance.

**Figure 5:**
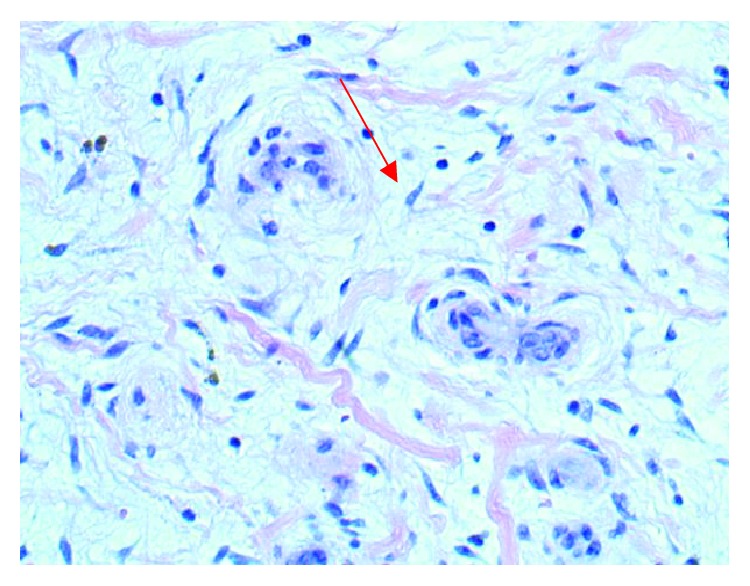
Red arrow identifying the presence of stellate cells.

Given the increased risk of embolization with mitral valve masses greater than 1 cm, we decided to undergo minimally invasive mitral valve excision and valve repair with P2 resection. Histopathological findings confirmed a 9.0 × 8.0 × 6.0 mm myxoma (Figure 3) attached to the external valve leaflet. The tumor was composed of stellate cells with eosinophilic cytoplasm, indistinct boarders, oval nucleus with open chromatin, and indistinct nuclei in the background of a myxoid substance (Figures 4 and 5). The patient's postoperative course was complicated by respiratory insufficiency likely related to obstructive sleep apnea which resolved within a few days following the procedure. Patient was discharged home with multidisciplinary outpatient follow-up.

## 3. Discussion

Primary cardiac tumors are rare with an incidence of less than one-tenth of a percent. In contrast, secondary cardiac tumors have been reported to be 20 times more common. The vast majority (>75%) of cardiac tumors are benign with the majority of those in adults being myxomas [3]. A meta-analysis which reviewed 1029 patients found that 83% of myxomas were located in the left atrial cavity and 12.7% were found within the right atrial cavity. Of these, it is estimated that 1–5% originate from the mitral valve leaflets [4]. As seen in our case, it is important to differentiate these masses from other valvular lesions as management would be vastly different. One must consider the possibility of infectious vegetation and papillary fibroelastoma.

Infective endocarditis is commonly associated with cardiac vegetations which appear as masses or flakes of various sizes. Typical echocardiographic findings associated with infective vegetations include localization on the upstream side of the valve, chaotic and orbiting motion independent of valve motion, and lobulated and amorphous shape. There may be associated abscess, pseudoaneurysm, paravalvular leak, or regurgitation [6–9].

Papillary fibroelastomas are valvular lesions typically located more commonly on the aortic valve but also may present on the other valve. These benign tumors are the second most common primary cardiac tumor in adults. Their appearance is compared to sea anemones as they have a characteristic central core with “arms” extending from the base [9]. On echocardiography, they appear as small, well-demarcated masses with uniform echodensity. These tumors may be round, oval, or irregular in appearance. The vast majority of these tumors are <20 mm in diameter. Nearly 50% of these tumors will have a stalk which may or may not be mobile [9–12].

The diagnosis of mitral valve myxomas is most commonly achieved with a combination of transthoracic and transesophageal echocardiography. These methods are relatively inexpensive, noninvasive, and widely available making them ideal in the initial evaluation of cardiac tumors. Echocardiography also provides an added benefit of assessing flow dynamics which may be altered based on the size of the cardiac mass. Cardiac MRI and CT can also provide useful information in assessing cardiac masses as they provide for much better resolution [13–16]. Histopathologic identification of stellate cells with eosinophilic cytoplasm and indistinct borders is diagnostic.

The treatment of mitral valve myxomas involves resection and, in our case, repair of the involved mitral valve. Our literature has also shown replacement of the mitral valve to be common practice based on the extent of involvement. Excision of the myxoma is recommended to avoid complications which include but are not limited to arrhythmias, heart failure, syncope, and embolism (tumor or thrombotic). Periodic reevaluation with echocardiography is recommended to assess for recurrence [15–18].
